# Is an Ideal Sense of Humor Gendered? A Cross-National Study

**DOI:** 10.3389/fpsyg.2018.00199

**Published:** 2018-02-27

**Authors:** Sümeyra Tosun, Nafiseh Faghihi, Jyotsna Vaid

**Affiliations:** ^1^Department of Psychology, University of Pretoria, Pretoria, South Africa; ^2^Department of Psychological and Brain Sciences, Texas A&M University, College Station, TX, United States

**Keywords:** sense of humor, ideal humor, everyday humor, gender, culture, creativity, sarcasm

## Abstract

To explore lay conceptions of characteristics of an ideal sense of humor as embodied in a known individual, our study examined elicited written narratives by male and female participants from three different countries of origin: United States, Iran, and Turkey. As reported in an earlier previous study with United States-based participants (Crawford and Gressley, 1991), our study also found that the embodiment of an ideal sense of humor was predominantly a male figure. This effect was more pronounced for male than for female participants but did not differ by country. Relative mention of specific humor characteristics differed by participant gender and by country of origin. Whereas all groups mentioned creativity most often as a component of an ideal sense of humor, this attribute was mentioned significantly more often by Americans than by the other two groups; hostility/sarcasm was also mentioned significantly more often by Americans than Turkish participants who mentioned it more often than Iranian participants. Caring was mentioned significantly more often by Americans and Iranians than by Turkish participants. These findings show a shared pattern of humor characteristics by gender but group differences in the relative prominence given to specific humor characteristics. Further work is needed to corroborate the group differences observed and to pinpoint their source.

## Introduction

There is an established literature on gender differences in humor perception and humor styles. Men have been noted to prefer humor that has sexual or aggressive themes whereas women appear to prefer neutral or absurd humor (Aillaud and Piolat, 2012). Whereas earlier studies showed that sexist humor (i.e., humor that upholds gender role stereotypes) is preferred over non-sexist humor (Cantor, 1976), other studies report that both men and women prefer humor that has the opposite gender as the butt (Vaid and Hull, 1998; Parekh, 1999). Furthermore, men typically rate themselves higher than women in humor initiation whereas women tend to rate themselves higher in humor appreciation, but when humor is studied in actual conversational contexts a more nuanced picture emerges (see Kramarae, 1981; Kotthoff, 1996, 2000; Schiau, 2017). Similarly, whereas some studies have found that humor produced by men is judged to be more humorous than that produced by women (Brodzinsky and Rubien, 1976), other studies have not found this effect (Hull et al., 2017), and still other work suggests a bias operating, whereby men are perceived to be the “funnier sex” regardless of how their humorous creations are actually judged (Mickes et al., 2011; Hooper et al., 2016).

Taken as a whole, the literature on gender and humor eludes easy generalization (see Martin, 2007, for a review). Methodologically, early studies have been criticized for their use of decontextualized or “canned” humor samples instead of spontaneously generated humor that arises naturally in conversation (Frecknall, 1994; Lampert and Ervin-Tripp, 1998). The literature on gender differences in humor preferences has also been critiqued for its reliance on classifications of humor type (as “hostile” or “sexual” or “sexist”) based on experimenter intuitions rather than eliciting participants’ own perceptions of humor type, which may not coincide with those of the experimenter (e.g., Parekh, 1999). Moreover, studies that have sought to measure the construct of a sense of humor have led to many promising instruments, such as the three Witz-Dimensionen (3WD) instrument by Ruch (1992), and to new adaptations of established instruments for use with non-English speakers (see Özdoğru, 2017). At the same time, it is recognized that for a construct as slippery and contextual as humor, it is important to consider multiple, converging measures across different groups and settings.

This recognition of the complexity of studying humor, together with a growing shift in regarding gender as performative, has led to a shift in humor scholarship in the direction of studying humor as it is enacted by men and women in a range of social contexts (e.g., Hay, 2000; Crawford, 2003), and in a range of laboratory contexts. Our own previous work has explored the relationship between cognitive, neurocognitive, and psycholinguistic aspects of humor detection and comprehension (e.g., Vaid, 2000; Vaid and Kobler, 2000; Vaid et al., 2003, 2015; Hull et al., 2005; Lopez and Vaid, 2017). Our work on humor production has sought to develop controlled ways of eliciting humor to study its cognitive and social underpinnings. For example, we developed a concept comparison task in which participants were asked to produce “catchy” ways in which the concepts were related, which invariably elicited humorous responses, e.g., MONEY and CHOCOLATE: *one swells the wallet, the other, the hips* (Hull et al., 2017). Another task involved generating rejoinders to proverbs, e.g., *Absence makes the heart grow fonder, but also makes the eyes wander* (Vaid, 2014). Other prior work in our laboratory has examined the role of culture in judgments about when humor (vs. silence) is an appropriate response to embarrassing situations encountered in daily life (Vaid et al., 2008). Finally, we have examined how individuals’ perceptions of their own humor styles compare with their perceptions of humor styles of members of their gender category and/or same or different cultural group (Quiros and Vaid, 1998; Vaid, 1999, 2006).

As an extension of our interest in gender and cultural dimensions of humor, the aim of the present research was to characterize how gender and country of origin (as a proxy for culture) may shape how individuals conceptualize an ideal sense of humor. The motivation for this study was a previous study which examined the role of gender in lay conceptions of an ideal sense of humor (Crawford and Gressley, 1991) in a large sample of United States-based participants of different ages and backgrounds. Participants in this study were asked to provide a brief narrative describing the humor characteristics of a person they knew who embodied an outstanding sense of humor. Crawford and Gressley (1991) reported that a majority of the participants identified a male figure as the person who embodied an outstanding sense of humor. Indeed, of the 141 respondents (49 men, 92 women), nearly 84% of men and 67% of women selected a male figure. The researchers also classified the humor characteristics mentioned into five categories: creativity (witty, clever, quick comeback), caring (humor used to put others at ease), real life (grounding the humor in real life experiences), jokes (having a repertoire of jokes), and hostility/sarcasm (satirical, biting humor) and noted that creativity, caring and real life were mentioned most often, and that there were no discernible differences in the weighting of these characteristics as a function of either participant gender or target gender.

Over 25 years have passed since the Crawford and Gressley (1991) study. While gender continues to be a salient element structuring society, women have also become more visible in a number of domains of public life, including in the realm of comedy. It is possible that gender stereotypes may have become less entrenched in the present day. We therefore wondered if the preference for a male figure as the embodiment of an outstanding sense of humor noted previously still holds among young adults in the present age. We also wondered whether individuals from other countries would show a similar preference, given that they might be less likely to be influenced by Western gender stereotypes (including stereotypes regarding men as being the canonical humor initiator), but might have their own cultural stereotypes about humor, gender, and the relation between the two. Although there have been a few prior studies of humor stereotypes in different nationalities, the focus of our study was on how individuals from the United States compared to those from two other countries in articulating characteristics of an ideal sense of humor, as embodied in someone they knew. Our interest was to uncover patterns of commonalities as well as differences across groups and across genders.

In searching the literature, we could find only one other empirical study conducted since the study by Crawford and Gressley (1991) that used their open-ended prompt. This study, by Nevo et al. (2001), was conducted on men and women in Singapore. It, too, found that the embodiment of an outstanding sense of humor was male. Of the 18 men and 46 women in the study, 76% of respondents selected a male target (Nevo et al., 2001). The researchers further noted that the preference for a male target was more pronounced in men, but no additional analyses were reported in terms of specific humor characteristics mentioned by men and women. Thus, we felt another study was warranted.

### The Present Research

Our study had two goals. The first was to investigate if the male preference first reported by Crawford and Gressley (1991) still holds. To examine this, we pooled data from United States-based college students tested from 2004 to the present. The second goal was to investigate if the pattern of a male preference as the embodiment of an ideal sense of humor is restricted to United States participants or is generalizable to other samples. In particular, we considered samples drawn from Iran and from Turkey, as these particular groups have been understudied in the humor literature; where country-based differences have been studied, they have either tended to be within north American/European samples or have compared Western with east Asian samples (e.g., China). Turkey is considered geographically and culturally as a bridge between Asia and Europe. Thus, we aimed to compare participants raised in a Western (American), a Middle Eastern (Iranian), and a blended (Turkish) culture. We did not have *a priori* expectations of how participants across the three groups would respond on the task; our study is exploratory with regard to the cultural dimension, as our sample sizes were limited and varied in other respects (e.g., age) and we recognize that much more follow up investigation would be needed to fully understand the nature of any differential patterns uncovered.

## Materials and Methods

### Participants

Participants included male and female United States born (American) and international students (born in Iran or in Turkey) recruited from a university town in the southwestern region of the United States, from a university in Istanbul, and from online responses. The American sample consisted of 279 undergraduate students (including 201 women) who ranged in age from 18 to 23 years, with a mean of 21 years. The majority self-identified as white, and the numbers of Latinx, African American, or Asian Americans were too few to permit separate subgroup analyses. The Iranian sample comprised 71 participants (47 women) who ranged in age from 17 to 54 years, with a mean age of 31.32 years, and the Turkish sample consisted of 79 undergraduate students (48 women) ranging in age from 18 to 25 years with a mean of 21.7. The American and Turkish participants completed the task as part of a class activity; the Iranian sample was recruited by placing an announcement in social media and participants completed an online version of the task. All participants received and answered the prompt in their primary language. The Iranian and Turkish data were translated into English by native Farsi- and Turkish speakers who had advanced English proficiency. Most of the data were coded by the same researcher (with gender of participants masked) to provide consistency in coding. A subset of the data were also intercoded to ensure some level of consensus (at least 80%).

### Materials and Procedure

Participants were given a response sheet on which they were to write a brief narrative in response to an open-ended prompt adapted from Crawford and Gressley’s (1991) study. They were instructed to think of a specific individual they knew who had an outstanding or ideal sense of humor and then to describe the characteristics of that humor, using three to five descriptors. They were then asked to describe the person who embodied that humor (we refer to this person as the *humor target*), indicating, for example, whether it was a family member, a friend, co-worker, or a comedian, and/or noting their gender, age, and ethnicity. There were no time constraints for responding. Since not all respondents stated the humor target’s gender, this information sometimes had to be inferred from the stated relation to the target (e.g., brother, sister, girlfriend, particular celebrity, etc.) or from the participants’ choice of pronouns in describing the person (however, this approach was helpful only for the English dataset as pronouns in Farsi and Turkish are not marked for gender).

### Data Analyses

Two sets of comparisons were conducted using chi square and regression analyses. The first examined percent mention of the target gender by participant gender and country. The second examined percent mention of each of the five categories of humor descriptors identified by Crawford and Gressley (1991) in relation to participant gender and country.

The five coding categories were as follows: **Creativity:** This characteristic includes terms referring to creative aspects of humor, like *witty, quick comeback, playing with language, clever*, as well as being spontaneous or natural. An example of this characteristic from our sample is “very quick in answering with a witty comment.” **Caring:** This characteristic indicates the kind of humor that makes people laugh and helps to change their mood when they are upset or in a tough situation. An example of this characteristic is “their humor helps relieve the tension.” **Real Life:** This characteristic shows the ability of the humorous person to tell stories and recount real life events in a humorous way. An example of this dimension is “a great story-teller to bring out humor.” **Jokes:** This characteristic refers to the use of actual jokes. An example of this dimension is “holds the crowd’s attention with a simple joke.” **Hostility/Sarcasm:** This category consists of attacking, insulting, and destructive humor as well as sarcasm. An indication of this characteristic is “can come up with the worst sexist insult.”

## Results

A summary of the relative distribution of target gender of the ideal humor person is provided in **Table 1** by participant gender and country of origin. Also included in the table are the number of participants per group for whom humor target gender was not specified. The latter comprised 21.81% of the American sample, none of the Turkish sample, and 59.15% of the Iranian sample.

**Table 1:** Gender distribution of humor target per participant gender and country.

Country of origin	Participant gender	Target gender		
		Female	Male	Unspecified
American	Female	45	118	38
	Male	5	50	23
	Total	50	168	61
Turkish	Female	15	33	0
	Male	2	29	0
	Total	17	62	0
Iranian	Female	6	13	28
	Male	3	7	14
	Total	9	20	42

### Identified Target Gender by Country of Origin

A chi square analysis was done excluding those whose target gender was unspecified to compare the relative percent mention of a male vs. female humor target, collapsed across participant gender. The analysis showed no significant effect of country of origin, χ^2^= 0.33, *p* = 0.85, *N* = 331. That is, regardless of their country of origin, participants showed a consistent tendency to select a male figure as their humor ideal: 77.1% of Americans, 78.5% of Turkish, and 73.5% of Iranian participants identified a male.

### Identified Target Gender by Country of Origin (American vs. Turkish vs. Iranian) and Participant Gender

**FIGURE 1:**
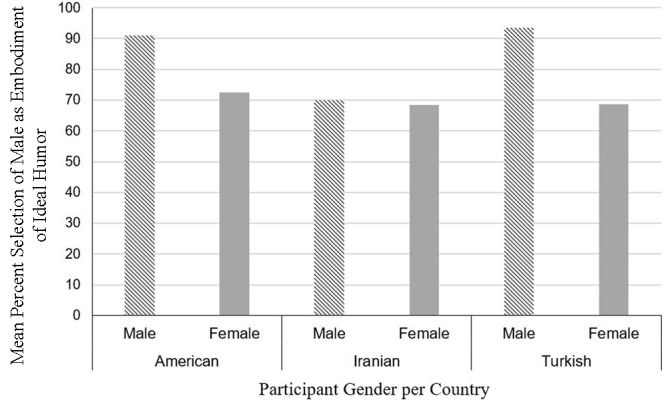
This figure demonstrates the relative mention of man humor target per participant gender and country (*only participants who specified the target gender*).

A logistic regression was conducted to see if the gender of the ideal humor target person could be predicted based on the participants’ gender or the participant’s country of origin (American, Turkish, Iranian). Again, only participants whose responses indicated the gender of their humor ideal were included in the analysis. Dummy coding was applied for the analysis. The model was significant, χ^2^= 13.78, *p* = 0.003, *df* = 3 and explained 6.2% of the variance. Gender of participant was a significant predictor of gender of humor target (χ^2^= 11.08, *p* = 0.001, *odds ratio* = 0.29): male participants were more likely than female participants to select a male target as the embodiment of an ideal sense of humor (89.6% vs. 71.9%, respectively). Country of origin, on the other hand, was not a significant predictor (χ^2^= 0.32, *p* = 0.85) (Turkish vs. Iranian: χ^2^= 0.13, *p* = 0.71, *odds ratio* = 1.19; American vs. Iranian: χ^2^= 0.31, *p* = 0.57; *odds ratio* = 0.94; American vs. Turkish: χ^2^= 0.04, *p* = 0.84, *odds* ratio = 0.79). See **Figure 1** for a depiction of the percent mention of male targets per participant gender and group.

### Identified Target Gender by Time Period and Participant Gender – American Sample Only

A logistic regression was conducted on the American sample to see if there was a difference related to time at testing in the percent mention of a male target by men and women. Here, Crawford and Gressley (1991) were compared with data from the American sample (which was collected over two different time periods, 2004 and 2014).

The model was significant, χ^2^= 14.97, *p* = 0.002, *df* = 3 and explained 6.9% of variance. There was not a difference between the American 2014 and the 1991 data. However, the American 2004 data showed a difference than both the 1991 data, χ^2^= 6.33, *p* = 0.012, *B* = -1.12 and the 2014 data, χ^2^= 4.88, *p* = 0.027, *B* = -1.01. Participants from the 2004 sample (89.6%) revealed more male favored results than the 2014 sample (73%) and than the original study sample (73%).

Participants’ gender was also a significant predictor, χ^2^= 5.29, *p* = 0.021, *B* = -0.767. That is, the selection of a male humor ideal was significantly higher when the participant was a male than when the participant was a female. In the original study male participants’ preference for a male target was 83.7% and female participants’ preference for a male target was 67.4%. In our study, male preference for a male target was 90.9% while female preference for a male target was 72.4%.

#### Analyses of Ascribed Humor Characteristics

An additional set of analyses was conducted on the influence of participant gender on relative mention of each of five characteristics of an ideal sense of humor. (A preliminary analysis that included target gender as an additional predictor yielded no effect of this variable and so we do not report it here.) **Table 2** provides a summary of the relative mention of each characteristic by male and female participants in each of the three groups. Note that these values represent all of the data per group, including those for whom target gender was not specified.

**Table 2:** Relative mention of each characteristic by each gender participant in each of the three groups.

Characteristic	Country	Male	Female
Creativity	Americans	56.4	66.7
	Iranians	45.8	44.7
	Turkish	45.2	50
Caring	Americans	32.1	38.3
	Iranians	12.5	29.8
	Turkish	3.2	2.1
Real life	Americans	21.8	32.3
	Iranians	12.5	27.7
	Turkish	9.7	2.1
Jokes	Americans	15.4	17.9
	Iranians	8.3	10.6
	Turkish	29	20.8
Hostility	Americans	35.9	42.8
	Iranians	0	0
	Turkish	12.9	22.9

*Numbers show percent mention*.

#### Humor Characteristics by Participant Gender and Country of Origin

**FIGURE 2:**
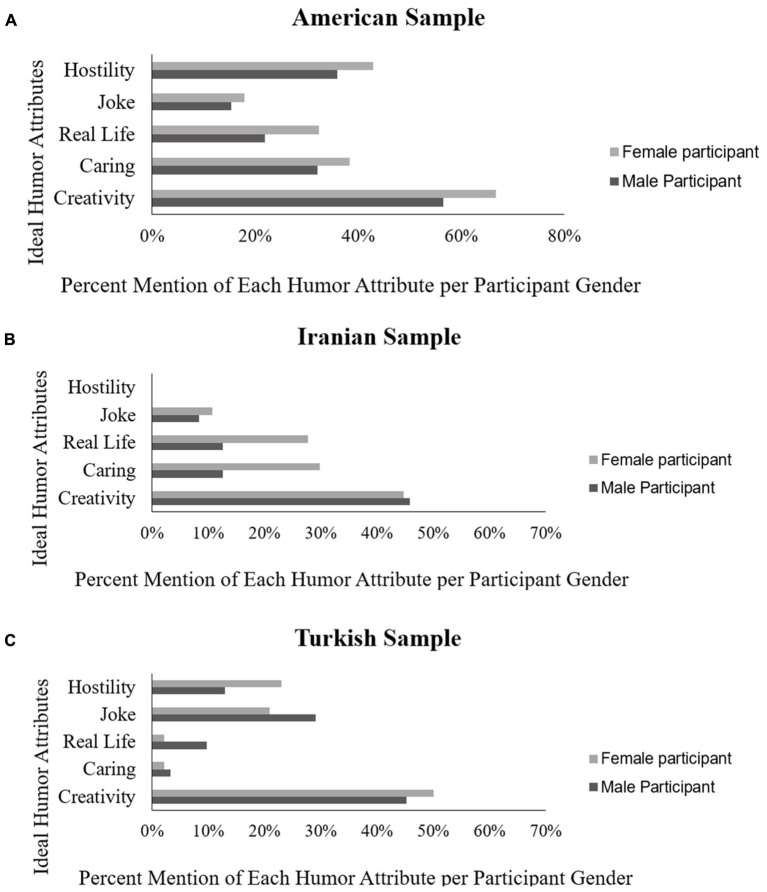
This figure demonstrates the relative mention of each characteristic by participant gender and country. **(A)** American sample; **(B)** Iranian sample; **(C)** Turkish sample.

Inspection of the relative percent mention of the five humor characteristics shows an overall predominance of mention of the creativity characteristic by men and women and across all groups. For Americans, the next most mentioned characteristic was hostility/sarcasm, followed by caring. For Iranians, by contrast, the order of mention of the five characteristics was: creativity, real life and caring, and for the Turkish sample, the order was creativity, joke and hostility/sarcasm (see **Figure 2**).

A multivariate regression analysis was conducted to jointly examine the effect of participant gender and country of origin (American, Turkish, and Iranian) with each of the five humor characteristics (creativity, caring, real life, jokes, and hostility/sarcasm) considered as separate dependent variables. The results demonstrated that overall, both participant gender (χ^2^= 2.67, *p* = 0.022, *df* = 5) and country of origin (χ^2^= 22.15, *p* < 0.001, *df* = 5) were significant predictors. However, no gender-specific effect was observed in any of the five humor characteristics. The results demonstrated that gender was a multivariate phenomenon, but gender did not specifically predict any of the five characteristics. Country of origin was a significant predictor for creativity (χ^2^= 10.30, *p* = 0.001, *R^2^* = 0.03, *odds ratio* = 0.905), caring (χ^2^= 13.39, *p* < 0.001, *R^2^* = 0.04, *odds ratio* = 0.902), and hostility (χ^2^= 54.85, *p* < 0.001, *R^2^* = 0.12, *odds ratio* = 0.816). Creativity was mentioned significantly more by American participants (64%) than by Turkish (48%) or Iranian participants (45%). Moreover, American (37%) and Iranian (34%) participants used caring to describe their ideal humor significantly more than did Turkish participants (2.5%). Further, hostility was mentioned significantly more by American participants (41%) followed by Turkish participants (19%) and it was mentioned least by Iranians (nearly 0%).

## Discussion

The aim of this study was to examine how men and women describe a specific person who embodies their ideal sense of humor. The study provided an opportunity to test whether the finding of a male target preference first noted by Crawford and Gressley (1991) for United States based participants and by Nevo et al. (2001) for Singaporean participants persists for Americans in the present period and is evident to the same extent among two other groups whose cultures are considered to be somewhat more traditional in terms of gender role stereotypes than American culture. Our findings show that the selection of a male as the embodiment of an ideal sense of humor was a pervasive and robust finding across the three samples we tested. Moreover, the size of this effect did not vary across the three groups. Of course, it is possible that the three samples we selected are on the gender inegalitarian end of the continuum and that had we selected a more egalitarian country we might not have found the effect. That remains for future work to test.

Our analysis of the United States samples tested at different periods of time further revealed that a male preference was actually somewhat stronger in the 2004 sample than it was for either the 1991 sample or a more recent 2014 sample. Perhaps the stronger male bias exhibited in the 2004 sample is a reflection of a public discourse in the country around that time regarding whether women can ever be as good at comedy as men. Nevertheless, it is interesting to note that, across all time periods sampled, the selection of a male target was significantly more likely when the participant was himself male. Thus, despite changes in societal consciousness about gender and humor that may have occurred (to differing degrees) over the past 25 years, there is a consistent preference for men to consider men as the embodiment of an ideal sense of humor. Moreover, this effect was found in the analysis by country of origin as well.

The finding that men are perceived as the embodiment of an ideal sense of humor may in part reflect an availability bias arising from the fact that male comedians and comedy writers still greatly outnumber female comedians and comedy writers. This difference in base rate may thus perpetuate a gender stereotype of men as the funnier sex and therefore prime people to think of men (rather than women) among their own acquaintances who exemplify an ideal sense of humor. Incidentally, among the American participants who provided information on their relationship to the gender target, a sizeable number (males and females) mentioned that the ideal humor person was their father. Further work should examine target gender demographic characteristics to provide insights into their relationship, if any, to the humor characteristics they embody.

Is there a difference in the types of characteristics used by men and women for male vs. female humor targets? Our analysis of the five dimensions noted by Crawford and Gressley (1991) to describe an ideal humor showed that the most frequently mentioned attribute by Americans in our sample is creativity, defined here as being witty, clever, and quick in coming up with a response. Creativity was mentioned by the majority of participants of both genders. The next most frequently mentioned dimensions for the American sample were hostility/sarcasm and caring. This may seem like an odd juxtaposition at first sight but it may not be that surprising given that the characteristic of “sarcasm” was coded under “hostility” and sarcasm (in American culture) is a way of interacting with one’s friends. Although the Turkish and Iranian samples also chose creativity most often as a defining characteristic of an ideal sense of humor, they differed from each other and from the American sample in other characteristics: caring was mentioned by the Iranian and the American samples to the same extent but was mentioned hardly at all by the Turkish participants. By contrast, hostility/sarcasm was mentioned hardly at all by the Iranian sample. We do not wish to over-interpret the particular group differences obtained, as we did not have *a priori* expectations. We present them here as descriptive data, in need of further exploration.

Our findings corroborate the overall pattern noted in the previous study by Crawford and Gressley (1991) on which the present research was based – namely, a preference for a male figure as the embodiment of an ideal sense of humor. However, the pattern of mention of the five different humor characteristics of the embodiment of an ideal sense of humor does not entirely concur with the pattern noted by Crawford and Gressley (1991). As already noted, there were some clear differences across the three cultural groups in the relative frequency of mention of some of the five humor characteristics.

Moreover, we recognize that our analysis of humor characteristics may also have been influenced in a substantive way by whether the target they were thinking of was male or female. As brought up by one of the reviewers of our article, it is possible that people tend to interpret or remember a given behavior differently depending on whether it comes from a man or a woman. Because of stereotypes or violation of expectations, a joke made by a woman could be interpreted as mean whereas the same joke made by a man could be interpreted as funny. Alternatively, participants could be selectively remembering humorous statements that conform to their gendered expectations of women as being caring, and thus describe the ideal humor for a female target as more caring. Unfortunately, given the paucity of female targets in our findings, we could not analyze humor characteristics as a function of target gender, but this is an important issue for further work.

Furthermore, we confined our analysis to only five humor categories based on those used in the previous study, a number of responses mentioned by participants in our study were not easily classifiable within that coding scheme. For example, a number of participants referred to “being able to laugh at themselves/take a joke” as being a valued characteristic in the person with an ideal sense of humor. Similarly, a number of respondents emphasized that the humor of this person was “inappropriate” but “not mean-spirited.” Or that it involved impersonation, funny facial expressions, etc. Based on these types of responses, it would be important in further work to do a more detailed content analysis than was possible in the present study. A need for additional investigation of this issue is underscored by our finding that a third of the Iranian sample’s narratives and from 5 to 10% of the American and Turkish narratives do not refer to any of the five characteristics of humor analyzed in this paper, and instead mention other characteristics of ideal humor that were not captured by these five categories. The use of political humor in everyday discourse, humor addressing tensions between the traditional vs. the modern (e.g., Apaydin, 2005), or individual differences in the relationship between humor and psychological well-being (e.g., Martin et al., 2003) would be interesting to explore in further work. Other approaches to measuring everyday humor (e.g., Craik et al., 1998) are also worth incorporating in future investigations of the role of gender and culture in the perception of an ideal sense of humor (see also Warren and McGraw, 2016).

Moreover, in addition to looking at humor type, it would be important to consider individuals’ age as another factor that will likely influence what is considered desirable in a sense of humor. As people grow older, they might prefer caring or wise humor over hostile humor. In this regard, it may be relevant to point out that the Iranian sample – which showed practically no mention of hostility/sarcasm – had a broader age range among participants than did the other two samples.

A further limitation of our study is that while the prompt was intentionally made open-ended, this made for some messiness in coding, as there were some terms, e.g, *inappropriate, crude or vulgar*, that we treated as interchangeable for the purpose of our coding, but which may not have been perceived by participants as synonyms. Similarly, a number of participants used *sarcastic* as an attribute, and in the present coding scheme it was coded as hostility, but it might not have been considered by participants to be a negative attribute. Of course, it is possible that an ideal sense of humor is perceived to include darker elements in addition to positive elements.

Another limitation of this study is that we classified participants and targets solely on the basis of their assigned gender, and thus cannot say anything about perceptions of humor by and about individuals whose gender assigned at birth does not coincide with their gender at the time of testing, or who consider themselves non-binary with regard to gender. Relatedly, since we did not administer any measures to assess participants’ gender identity or gender role attitudes, our data do not allow us to say anything about how participants’ attitudes toward feminism or traditional gender roles may have informed their responses. Similarly, a gendered humorous persona might not be considered appealing to some participants in this “gender fluid” era.

These limitations notwithstanding, our study, using an implicit, elicited measure of lay conceptions of an ideal sense of humor, allows us to conclude that, in highly gender inegalitarian societies, the ideal sense of humor is strongly gendered in favor of male targets, especially among men. Further, this male preference is as firmly entrenched in contemporary American culture (at least for the young adult age range sampled in the present research) now as it was nearly 25 years ago. Third, a bias for thinking of men as the embodiment of an ideal sense of humor is not restricted to those in an American cultural context but is also found among members of two other nationalities. What is important to note is that these groups, despite being considered more “traditional,” nevertheless did not show a stronger gender effect than that observed in the American sample. Finally, our results indicate that across all groups the most salient dimension of an ideal sense of humor is the ability to be witty, creative, and quick; this dimension is most pronounced for the American sample, who also appeared to value hostility/sarcasm and caring. The Iranian sample in turn appeared to value real life humor and caring, whereas the Turkish group placed least value on caring but instead emphasized jokes and hostility/sarcasm.

In further work it will be important to probe deeper into the social context of humor use in everyday life to determine not just what the characteristics are of an ideal sense of humor in an abstract sense, but how those characteristics are brought to life in different kinds of interactions.

## Ethics Statement

This study was carried out in accordance with the recommendations of the Institutional Review Board guidelines with written informed consent from all subjects. All subjects gave written informed consent in accordance with the Declaration of Helsinki. The protocol was approved by the Texas A&M University Institutional Review Board Committee.

## Author Contributions

JV and ST contributed to the design of experimental stimuli and procedure. All authors contributed to the manuscript in terms of conception, implementation of experimental protocols, data collection and analysis, and editing of drafts.

## Conflict of Interest Statement

The authors declare that the research was conducted in the absence of any commercial or financial relationships that could be construed as a potential conflict of interest.
